# Supplementary material for: AffyMAPSDetector: a software tool to characterize Affymetrix GeneChip™ expression arrays with respect to SNPs
Source: BMC Bioinformatics. 2007 Jul 30;8:276. doi: 10.1186/1471-2105-8-276 (PMC1959249; doi:10.1186/1471-2105-8-276)
Supplement: Additional file 13 — AffyMAPSDetector v1 source code. AffyMAPSDetector source code. [file 1471-2105-8-276-S13.zip › AffyMAPSDetector_v1_src/doc/index-files/index-15.html]

P-Index


|  |  |  |  |
| --- | --- | --- | --- |
| **Overview** \| Package \| Class \| Use \| **Tree** \| **Deprecated** \| **Index** \| **Help** | | |  |
| **PREV LETTER**   **NEXT LETTER** | **FRAMES**    **NO FRAMES**     **All Classes** |  |  |


A B C D E F G H I J L M N O P R S T V W X Y 

---


## **P**

**PM\_MM\_RATIO\_DATA** - Static variable in class affychip.snpdetector.Constant: **PROBE\_INFO\_FILE** - Static variable in class affychip.snpdetector.Constant: **PROBE\_INFO\_FILE\_SEPARATOR** - Static variable in class affychip.snpdetector.Constant: **PROBE\_INFO\_HEADERS** - Static variable in class affychip.snpdetector.Constant: **PROBE\_INFO\_PROBESET\_ID\_FIELD\_INDEX** - Static variable in class affychip.snpdetector.Constant: **PROBE\_SEQ\_FIELD\_INDEX** - Static variable in class affychip.snpdetector.Constant: **PmMmHeatMapViewer** - class affychip.snpdetector.util.PmMmHeatMapViewer.: Title: Probe SNP Detector **PmMmHeatMapViewer()** - Constructor for class affychip.snpdetector.util.PmMmHeatMapViewer: **PmMmHeatmapViewerModel** - class affychip.snpdetector.util.PmMmHeatmapViewerModel.: Title: Probe SNP Detector **PmMmHeatmapViewerModel()** - Constructor for class affychip.snpdetector.util.PmMmHeatmapViewerModel: **PmMmHeatmapViewerModel(String)** - Constructor for class affychip.snpdetector.util.PmMmHeatmapViewerModel: **PmMmHeatmapViewerModel(File)** - Constructor for class affychip.snpdetector.util.PmMmHeatmapViewerModel: **PmMmHeatmapViewerModel.Row** - class affychip.snpdetector.util.PmMmHeatmapViewerModel.Row.: Title: Probe SNP Detector **PmMmHeatmapViewerModel.RowComparatorForDataSorting** - class affychip.snpdetector.util.PmMmHeatmapViewerModel.RowComparatorForDataSorting.: **PmMmHeatmapViewerModel.RowComparatorForDataSorting()** - Constructor for class affychip.snpdetector.util.PmMmHeatmapViewerModel.RowComparatorForDataSorting: **PmMmMatrixLoader** - class affychip.snpdetector.util.PmMmMatrixLoader.: Title: Probe SNP Detector **PmMmMatrixLoader(String)** - Constructor for class affychip.snpdetector.util.PmMmMatrixLoader: **PmMmMatrixLoader(File)** - Constructor for class affychip.snpdetector.util.PmMmMatrixLoader: **Probe** - class affychip.snpdetector.model.Probe.: Title: Probe SNP Detector **Probe(String, String, String, String, int)** - Constructor for class affychip.snpdetector.model.Probe: **ProbeSet** - class affychip.snpdetector.model.ProbeSet.: Title: Probe SNP Detector **ProbeSet(String)** - Constructor for class affychip.snpdetector.model.ProbeSet: **parseProbe(String)** - Static method in class affychip.snpdetector.model.SnpFinder: probe:HG-U95Av2:1000\_at:399:559; Interrogation\_Position=1367; Antisense;TCTCCTTTGCTGAGGCCTCCAGCTT **pmMmEqParam** - Static variable in class affychip.snpdetector.util.PmMmHeatMapViewer: **probeSNPs(String)** - Method in class affychip.snpdetector.model.ProbeSet: **probeSNPs(String[])** - Method in class affychip.snpdetector.model.ProbeSet

---


|  |  |  |  |
| --- | --- | --- | --- |
| **Overview** \| Package \| Class \| Use \| **Tree** \| **Deprecated** \| **Index** \| **Help** | | |  |
| **PREV LETTER**   **NEXT LETTER** | **FRAMES**    **NO FRAMES**     **All Classes** |  |  |


A B C D E F G H I J L M N O P R S T V W X Y 

---
